# The impact of the federal menu labeling law on the sentiment of Twitter discussions about restaurants and food retailers: An interrupted time series analysis

**DOI:** 10.1016/j.pmedr.2023.102478

**Published:** 2023-10-14

**Authors:** Yulin Hswen, Alyssa J. Moran, Tayla von Ash, Siona Prasad, Tarun Martheswaran, Denise Simon, Lauren P. Cleveland, John S. Brownstein, Jason P. Block

**Affiliations:** a Department of Epidemiology and Biostatistics, University of California San Francisco, CA, USA; b Bakar Computational Health Sciences Institute, University of California San Francisco, CA, USA; c Department of Health Policy and Management, Bloomberg School of Public Health, Johns Hopkins University, Baltimore, MD, USA; d Behavioral and Social Sciences, Brown University, Providence, RI, USA; e Harvard University, Cambridge, MA, USA; f Stanford University, Palo Alto, CA, USA; g Division of Chronic Disease Research Across the Lifecourse (CoRAL), Department of Population Medicine, Harvard Medical School and Harvard Pilgrim Health Care Institute, Boston, MA, USA.; h Computational Epidemiology Lab, Harvard Medical School, Boston, MA, USA

**Keywords:** Calorie, Menu labeling law, Twitter, Health policy, Sentiment analysis, Public opinion

## Abstract

The US federal menu labeling law, implemented on May 7 th 2018, required that restaurant chains post calorie counts on menu items. The purpose of this study was to analyze the change in public sentiment, using Twitter data, regarding eight restaurant chains before and after the calorie labeling law’s implementation. Twitter data was mined from Twitter’s application programming interface (API) for this study from the calendar year 2018; 2016 and was collected as a control. We selected restaurant chains that had a range of compliance dates with the law. Tweets about each chain were filtered by brand-specific keywords, and Valence Aware Dictionary and sEntiment Reasoner (VADER) sentiment analysis was applied to receive a continuous compound score (−1–1) of how positive (1) or negative (−1) each tweet was. Controlled Interrupted Time Series (CITS) was performed with Ordinary Least Squares (OLS) Regression on 2018 and 2016 series of compound scores for each brand, and level and trend changes were calculated. Most restaurant chains that implemented the federal menu calorie labeling law experienced no change or a small change in level or trend in sentiment after they implemented labeling. Chains experienced mildly more negative sentiment right after the law was implemented, with attenuation of this effect over time. Calorie labeling did not have a strong effect on the public’s perception of food brands over the long-term on Twitter and may imply the need for greater efforts to change the sentiment towards unhealthy restaurant chains.

## Highlights

• Changes in public sentiment towards restaurant chains were monitored by Twitter.
• Calorie Labeling had a short-term impact on sentiment toward restaurant chains.
• Negative sentiment toward restaurant chains after calorie labeling attenuates over time.
• Public sentiment toward restaurant chains does not change over the long term.
• More efforts to change the sentiment toward unhealthy restaurant chains are needed.

## 1 Introduction

Americans are eating more meals outside of the home, including at sit-down and fast-food restaurants. American adults consume an estimated 34 % of calories outside of the home, with 11 % of calories consumed from fast-food (Fryar et al., 2018). Because food eaten outside of the home has higher energy density and fat content and is offered in larger portion sizes than food consumed at home, this trend in away-from-home food consumption has contributed to worsening dietary quality and health outcomes. Fast-food meals, in particular, also contain relatively few fruits and vegetables and are inexpensive and ubiquitous.

Food policy has the potential to influence individuals’ eating decisions and behaviors on a large scale. Examples of food policy legislation intended to improve diet include soda taxes, regulating portion sizes in restaurants, nutrition education, and labeling nutrition content on prepared food in restaurants in supermarkets.

As part of the 2010 Affordable Care Act, the US Food and Drug Administration required restaurant chains, with 20 or more locations, to disclose the number of calories in each menu item on menus and menu boards (Food and Drug Administration, HHS, 2014). The goal of this policy, which restaurants were required to comply with by May 7, 2018, was to make nutrition information easily accessible at the point of purchase to better enable informed (and healthy) consumer dietary choices. A key driver for this regulation was that consumers have a general lack of awareness of the calorie content of restaurant foods; making this information more accessible might lead consumers to make alternate (and healthier) choices (Food and Drug Administration, HHS, 2014).

Studies of the effectiveness of menu labeling on improving food choices and consumption are mixed (Petimar et al., 2021). Studies are needed to examine how menu labeling might influence not only eating behaviors but perceptions. In the proposed study we used sentiment analysis of consumer social media data from Twitter to examine whether the menu labeling law resulted in a shift in consumer sentiment. We hypothesized that public/consumer sentiment towards restaurants and food retailers would become more negative after implementing menu labeling.

## 2 Materials and methods

### 2.1 Data collection

Data were collected from the Twitter Application Programming Interface (API) which allows for the collection of publicly available data that represent approximately 1 % of all Twitter data (developer.twitter.com). Through the API connection, tweet data for 2016 and 2018 were collected with geocoordinate parameters restricted to tweets with latitude and longitudinal coordinates located within the United States. Data from 2018 were considered to cover the intervention period; 2016 was used as a control year. 2017 data were not used in this analysis, as the labeling law was originally scheduled for May 2017, but moved to May 2018. Tweets were then subsequently filtered for each restaurant by relevant keywords and subsequently analyzed.

Unexpected missing data appeared with the API, due to slow processing and other common errors. This caused some brands to have no data on certain days throughout the year, and in these cases, linear interpolation was used to fill in missing values.

### 2.2 Restaurant chains and federal label law compliance

Because the calorie labeling policy was delayed, several chains implemented labeling in advance of the May 2018 deadline (Block, 2018). We captured data on chains that met several categories of labeling compliance: Early adopters (complied before the May 2018 implementation date; on time (complied by May 2018); and non-compliant (did not comply in 2018) (Block, 2018). Within these categories, we selected chains across three categories of restaurants: pizza, fast food, and full service/sit-down (Table 1).

**Table 1:** Categories of restaurants by timing of calorie labeling implementation: Pizza, Fast Food, and Full Service.

**Category**	**Early Adopter (prior to 2018)**	**On Time (May 2018)**	**Non-Compliant**
Pizza	Pizza Hut	Papa John’s	Domino’s
Fast Food	McDonald’s	Burger King	Five Guys
Full Service	Chili’s	Olive Garden	Uncle Julio’s

### 2.3 Sentiment analysis

Valence Aware Dictionary and Sentiment Reasoner (VADER) (Elbagir and Yang, 2019) is a tool specifically designed to analyze social media phrasing. VADER sentimental analysis is based on a lexicon dictionary that maps the polarity of sentiment (positive, negative, neutral) of a speaker or writer based on the emotion of the lexical (vocabulary, language) features. For this study, VADER was used to perform sentiment analyses on tweets containing keywords related to each restaurant chain. The sentiment score for each tweet string was calculated by summing sentiment for each word within the VADER dictionary and averaging it for the sentiment for each tweet; Twitter handles and special characters were removed and did not contribute to scores. Further we normalized the value of the tweet with the following:

$$\frac{x}{\sqrt{x^{2}+\alpha }}$$

where x is the sum of all sentiment scores of words within the tweet, while α is a normalization parameter. VADER also takes into account different punctuations when calculating a compound sentiment score, as the string score is amplified with exclamation marks relative to periods. Factors such as capitalization and common “booster” words also have an impact on the compound score. A continuous compound polarity score was calculated for each full tweet between −1 to +1. According to standard sentiment scoring practice, scores that were <−0.5 were considered negative while >0.5 were positive and between −0.5 to +0.5 were considered netural (Elbagir and Yang, 2019).

### 2.4 Statistical analyses

Data from VADER Sentiment was obtained by averaging compound values by day, to maximize data points. A locally weighted scatterplot smoothing plot, locally weighted scatterplot smoothing (lowess), was used to reduce seasonal fluctuations and served to better detect trend changes related to the policy change (Royston, 1992). A controlled interrupted time series analysis was used to compare compound sentiment about restaurant chains before and after the May 7th policy implementation date, which is considered the “intervention” of interest for this analysis. The ‘trend” change estimates the change in the slope of compound sentiment scores after the implementation date while the “level” change measures the immediate intervention effect (Cawley et al., 2021).

The resulting regression for the time series becomes:

(1) Y_with policy_ = β_0_ + β_1_*time + β_2_*policy + β_3_*time_after_policy

β_0_ is the mean compound sentiment per day on day 1 of the year, β_1_ is the daily **trend** change in mean compound sentiment before the intervention, β_2_ is the **level** change in mean compound sentiment after May 7, 2018 (control 2016), and β_3_ is the change in the trend of mean sentiment after the intervention, relative to the trend before. Sensitivity analyses were conducted with and without removing outliers through the z-score filter technique (Rousseeuw and Hubert, 2011). Our outcome variable Y designates the compound sentiment score, after lowess smoothing. For our interrupted time series analysis, we calculate two values of this outcome variable. Y_with policy_ comes from Equation 1, and is a reflection of estimated compound sentiment with the nutrition policy in place. Y_without policy_ comes from Equation 2, and represents the estimated compound sentiment without the policy, based on a subset of policy-invariant coefficients from Model 1.

(2) Y_without policy_ = β_0_ + β_1_*time

To express intervention effects, we calculated a percentage change in outcome due to the intervention calculated as

(3) 100(Y_with policy_- Y_without policy_)/Y_without policy_

All percentages were calculated with point values from the last day of 2018 (control for 2016), such as the example below with tweets about Pizza Hut pizza sentiment values. Assume from our linear regression models, that we obtained coefficient values of β_0=_0.0767, β_1=_0.0003, β_2=_-0.0228, and β_3=_-0.0004. Then, as there are 365 days in the year 2018, and the policy was implemented on day 127 of 2018, our “time_after policy” value is 365–127 = 238.

Y_with policy=_ 0.0767 + 0.0003 (365) −0.0228(1)-0.0004(238) = 0.0682

Y_without policy=_ 0.0767 + 0.0003 (365) = 0.1862

The resulting percentage, using equation (2) is −63.4 %.

All regression and time series analyses were performed separately using R 4.11 software for 2016 as the control and 2018 as the policy/intervention period. Coefficient significance for was set at p ≤ 0.05.

## 3 Results

Findings yielded changes in level and/or trend for several restaurant chains, but with small absolute magnitude change (Table 2).

**Table 2:** Level and Trend coefficient changes by Restaurant Chain, before and after policy.

Restaurant Chain	Level Change 2016	Trend Change 2016	Level Change 2018	Trend Change 2018
**Pizza Hut**	−0.0094 (p = 0.327)	−0.0001 (p = 0.311)	**−0.0228** **(p = 0.001)**	**−0.0004** **(p < 0.001)**
**Papa John’s**	−0.0090 (p = 0.309)	−0.0001 (p = 0.209)	0.0032 (p = 0.725)	**0.0005** **(p < 0.001)**
**Domino’s**	−0.0031 (p = 0.435)	**0.0002** **(p < 0.001)**	0.0059 (p = 0.055)	−8.066e-05 (p = 0.058)
McDonald’s	**−0.0119** **(p < 0.001)**	−6.371e-06 (p = 0.836)	**−0.0078** **(p = 0.004)**	**0.0001** **(p = 0.002)**
**Burger King**	**−0.0213** **(p < 0.001)**	**0.0002** **(p = 0.003)**	−0.0139 (p = 0.127)	**−0.0004** **(p = 0.005)**
Five Guys	**0.0370** **(p = 0.014)**	−8.729e-05 (p = 0.625)	−0.0035 (p = 0.578)	2.844e-05 (p = 0.741)
**Chili’s**	0.0188 (p = 0.079)	0.0001 (p = 0.360)	**−0.0450** **(p < 0.000)**	−0.0002 (p = 0.117)
**Olive Garden**	**0.0309** **(p = 0.001)**	**0.0004** **(p < 0.001)**	−0.0112 (p = 0.059)	6.069e-05 (p = 0.459)
Uncle Julio’s	0.0188 (p = 0.692)	**−0.0027** **(p < 0.001)**	0.0040 (p = 0.925)	0.0007 (p = 0.249)

^1^ Each cell contains a coefficient as well as the p-value for regression. Bolded cells represent significance.

### 3.1 Main analysis

#### 3.1.1 2016

Twitter discussions reveal sentiment changes, as determined by VADER sentiment analysis, that are correlated with the same calendar date on which calorie labeling was implemented in 2018 (Food and Drug Administration, HHS, 2014). Increases in positive sentiment were represented by positive value parameter estimates and percent changes; increases in negative sentiment were represented by negative value parameter estimates and percent changes. Percent changes represent the expected percent change in sentiment on the last day of the year. Among pizza chains, in 2016, Pizza Hut and Papa John’s experienced no significant changes in level (β_2)_ and trend (β_1)_ after May 7, 2016, while Domino’s experienced a significant change in trend (0.0002, p < 0.001) and 96.5 % change in average sentiment score. Among fast food chains, McDonald’s faced a significant change in level (−0.119, p < 0.001) and −11.9 % change in average sentiment score. Burger King experienced a significant change in both levels (−0.0213, p < 0.001) and trend (0.0002, p = 0.003), as well as a 48.0 % change in average sentiment score. Five Guys experienced only a significant change in level (0.0370, p = 0.014). Among full-service chains, Chili’s experienced no changes in level and trend. Olive Garden saw a significant change in both levels (0.0309, p = 0.001) and trend (0.0004, p < 0.001), as well as 1106.1 % change in sentiment. Lastly, Uncle Julio’s experienced a significant change in trend (−0.0027, p < 0.001) and −82.9 % change in average sentiment (Results in Table 2).

#### 3.1.2 2018

In 2018, Pizza Hut displayed a significant change in both level (−0.0228, p = 0.001) and trend (−0.0004, p < 0.001), with a −63.4 % change in average sentiment on the last day of 2018. Papa John’s experienced a significant change in trend (0.0005, p < 0.001), and −362.6 % change in sentiment. Domino’s had no significant change in level and trend. McDonald’s experienced a significant change in level (−0.0078, p = 0.004) and trend (0.0001, p = 0.002), as well as a 25.9 % change in sentiment. Both Burger King and Five Guys experienced no significant changes in 2018. Five Guys experienced no significant changes in level or trend during 2018. Chili’s experienced a significant change in level (−0.0450, p < 0.001), with −30.6 % change in average sentiment and no change in trend. Olive Garden experienced no significant changes in level and trend in 2018.

### 3.2 Sensitivity analysis

Using z-score outlier elimination (Rousseeuw and Hubert, 2011), the results for the repeated Controlled-Interrupted time series analysis are described below for 2016 and 2018. Results are provided in the Appendix. No distinct patterns of difference were seen as a result of the federal labeling law on the sentiment of food brands in 2016 or 2018 from these sensitivity analyses.

## 4 Discussion

In this study, we assessed changes in public sentiment toward large U.S. restaurant chains after the implementation of the federal calorie menu labeling law by analyzing discourse on Twitter. Contrary to our hypothesis, we found no consistent change in sentiment towards chains immediately following the implementation of the law. We detected small changes in trends in sentiment towards restaurants in the post-implementation period, but changes were variable across chains. For example, sentiments towards Papa John’s, a pizza restaurant, grew more positive in the post-implementation period, but sentiments towards Burger King, a fast-food restaurant, grew more negative. There were no changes in sentiments towards Olive Garden, a full-service Italian restaurant.

These findings could be explained by the slow roll-out of menu labeling, which may have impacted consumer responses and restaurant practices prior to the final implementation deadline in May 2018. Many restaurants posted calorie labels on their menus prior to the deadline, with some restaurants, like McDonald’s, posting calories nationwide as early as 2012 (Petimar et al., 2019, Block, 2018, Cleveland et al., 2020 Apr). Data from New York City, which required chain restaurants operating 15 or more locations in New York City to post calories starting in 2008, suggest that consumer awareness of calorie labels prompted by a law or regulation declines over time. In one study, only 37 % of restaurant diners reported noticing calorie labels five years after the policy went into effect compared to 51 % immediately after the policy was implemented (Cantor et al., 2015). Additionally, many national chain restaurants made changes to reduce calories in their menu items during the lead-up to the national implementation deadline. Between 2012 and 2018, the 66 top-revenue generating national chains removed some high-calorie items from their menus and introduced new, lower-calorie items (Bleich et al., 2018, Bleich et al., 2012–2018.). Some of these changes in advance of the law might have limited consumers’ recognition of changes after the law officially went into effect.

Studying public sentiment towards restaurants in the wake of the menu labeling legislation can help explain the extent to which calorie labels increased public awareness of calories in restaurant food. Increasing public awareness was a primary goal of the menu labeling legislation, but research on how effectively the legislation achieved that aim is mixed. Prior to menu labeling, consumers significantly underestimated calories in restaurant meals (Fryar et al., 2018, Food and Drug Administration, HHS, 2014, Block, 2018, Elbagir and Yang, 2019, Rousseeuw and Hubert, 2011, Royston, 1992, Rao and Srivastava, 2012, Petimar et al., 2021, Block et al., 2013 May, Cawley et al., 2021, Elbel, 2011 Oct, Roberto et al., 2010). A 2010–2011 study in 89 fast-food restaurants in the Northeast U.S. found that adults and adolescents underestimated calories in meals purchased by 21 % and 34 %, respectively, prior to labeling (Block et al., 2013). Some studies have shown that disclosing calorie content at point-of-purchase can improve consumer knowledge of calories, but overall knowledge remains low, even with labeling (Cawley et al., 2021, Elbel, 2011 Oct, Roberto et al., 2010). A randomized controlled experiment of calorie labels on menus of a full-service restaurant found that labels improved the accuracy of consumers’ post-meal calorie estimates by 4 %, with still a large margin of error (34.2 %) after labeling.^3^ Some research on the effects of labeling in a real-world setting has shown positive effects. An evaluation of the New York City menu labeling law found that the number of consumers accurately estimating calories in their fast-food meals increased from 15 % before labeling to 24 % after labeling (Elbel, 2011). A study of social media discourse found an increasing trend in Tweets related to calories after the implementation of the federal law; this occurred alongside a decreasing trend in Google searches for calories after the implementation of the federal menu labeling law, which may be an indication of growing awareness of the calorie labels and thus less need for searches (Hswen et al., 2021). Despite some indications of rising awareness, recognition of calories remains low (Kiszko et al., 2014).

Theoretically, if calorie menu labels increase awareness, consumer satisfaction with restaurant menu items may change, particularly for items that are unexpectedly high or low in calories. Originating in the marketing literature, expectancy disconfirmation theory posits that customer satisfaction with a product is a function of both the quality of the product and the extent to which the product matches what the customer expected (Oliver, 1980). A large body of research has shown that when a product does not meet expectations, satisfaction decreases; when a product exceeds expectations, satisfaction increases (Syzmanski and Henard, 2001). Although labels likely work through more than one decision-making pathway, some prior work has shown greater effects of labels on consumer choice when the labels provide information that deviates from the consumer’s initial expectations. For example, an online randomized experiment of warning labels on sugary drinks found that labels reduced parents’ selection of fruit drinks, which most consumers rated as “healthy”, but did not change the selection of soda, which most rated as “unhealthy” prior to viewing labels (Moran and Roberto, 2018). Based on this theory, calorie labels would be most influential when placed on products for which consumers have false assumptions about dietary quality. The labels could elicit positive or negative reactions from consumers, depending on the direction of the deviation. For example, consumers may be pleased to find that fast-food items, often considered to be “unhealthy,” have fewer calories than expected; they may be disappointed to find that an item typically viewed as “healthy,” such as a salad from a full-service restaurant, is much higher in calories than anticipated (Joe et al., 2020).

From a public health perspective, the impact of menu labeling on customer satisfaction is important. If customers express negative reactions to the calorie content of foods, it may encourage restaurants to reformulate existing menu items to be lower in calories, or to introduce new, lower calorie items. Restaurant customers actively engage with their peers through social media platforms, such as Twitter, to advise on purchase decisions, and restaurants commonly use social media to monitor customer sentiment and solicit feedback on menu items (Intouch insight, 2019, Mhlanga and Tichaawa, 2017). Our results showed a brief change in sentiment towards chains after calorie labeling that was not sustained, indicating that the simple provision of calorie information may not have the power to modify eating behavior and influence healthier food choices. Thus, with fairly limited change in sentiment after labeling, chains might be reassured that changes they have made do not have a meaningful negative effect on discussions about them.

## 5 Strengths and limitations

This study has several limitations. First, calorie labeling implementation varied across chains. Some of the effects of labeling on sentiment regarding chains might have been more pronounced around the exact time of labeling. Because we used the national implementation date as the intervention date, this may have underestimated the effects of chains that implemented labeling before or after that date. The delays in the calorie labeling law also might have blunted its effect; customers had longer to prepare for it, or they may have ignored the final compliance date because it changed so many times (the law passed a full eight years prior/ to implementation). Second, we could not determine the source of tweets. Restaurant chains might mention their chains frequently on Twitter, with standardly positive sentiment. Chains might have increased their traffic around the time of labeling, leading to an overestimate of positive sentiment after.

As for strengths, this study utilized a large social media database to examine the effect of labeling on sentiment regarding some of the top-selling restaurant chains in the United States. No prior studies have assessed the effect of labeling on public sentiment regarding chains, and few studies have used these methods for measuring public sentiment regarding nutrition policy interventions.

## 6 Conclusions

The effectiveness of nutrition policies in improving dietary quality relies on the public’s understanding of what these labels mean, what constitutes healthy food, and psychological factors that would lead them to make healthy decisions in the face of such information.

There were minimal changes in public sentiment towards U.S. chain restaurants following the federal menu labeling legislation. Organizations that seek to amplify the effects of nutrition policies may need greater attention and could use social media to help bring attention to the positive effects these laws could bring.

## Funding

Funding for this research was provided by the National Heart, Lung, and Blood Institute (T32HL098048).

## 8 Institutional review board statement

Ethical review and approval were waived for this study as this study was considered non-human subjects research.

## CRediT authorship contribution statement

**Yulin Hswen:** Conceptualization, Methodology, Investigation, Resources, Data curation, Writing – original draft, Supervision, Project administration. **Alyssa J. Moran:** Writing – original draft, Methodology. **Tayla von Ash:** Writing – original draft, Methodology. **Siona Prasad:** Formal analysis, Investigation. **Tarun Martheswaran:** Formal analysis, Investigation. **Denise Simon:** Project administration. **Lauren P. Cleveland:** Project administration. **John S. Brownstein:** Supervision, Resources, Funding acquisition. **Jason P. Block:** Supervision, Resources, Funding acquisition, Conceptualization, Writing – review & editing.

## Declaration of Competing Interest

The authors declare that they have no known competing financial interests or personal relationships that could have appeared to influence the work reported in this paper.

## Figures and Tables

**Table A1:** Regression results after omitting outliers from original time series data (see Table 1).

Food Item	Level Change 2016	Trend Change 2016	Level Change 2018	Trend Change 2018
**Pizza Hut**	0.0116 (p = 0.327)	−0.0001 (p = 0.311)	**−0.01669** **(p = 0.009)**	−0.00013 (p = 0.146)
**Papa Johns**	−0.0075 (p = 0.344)	**−0.0002** **(p = 0.029)**	0.00482 (p = 0.594)	**0.0005** **(p < 0.001)**
**Dominos**	0.0022 (p = 0.494)	**0.0002** **(p < 0.001)**	**0.0099** **(p < 0.001)**	−2.8e-05 (p = 0.483)
McDonalds	**−0.0060** **(p = 0.005)**	−3.8e −06 (p = 0.141)	**−0.0067** **(p = 0.008)**	**0.0001** **(p < 0.001)**
**Burger King**	**−0.0160** **(p < 0.001)**	**0.0001** **(p < 0.001)**	**−0.0130** **(p = 0.004)**	−4.9e-05 (p = 0.437)
Five Guys	−0.0030 (p = 0.775)	−0.0002 (p = 0.055)	**0.0110** **(p = 0.047)**	**0.0001** **(p = 0.014)**
**Chili’s**	0.0041 (p = 0.612)	**0.0002** **(p = 0.032)**	**−0.0585** **(p < 0.001)**	**−0.0004** **(p = 0.001)**
**Olive Garden**	0.0027 (p = 0.742)	−1.4e-05 (p = 0.885)	**−0.0166** **(p = 0.005)**	6.9e-05 (p = 0.932)
Uncle Julio’s	0.0188 (p = 0.692)	**−0.0027** **(p < 0.001)**	−0.0212 (p = 0.619)	0.0006 (p = 0.339)

^2^ Each cell contains coefficient as well as p-value for regression. Bolded cells represent significance.

## Data Availability

The authors do not have permission to share data.

## Appendix A

After omission of outliers for 2016 time series, Pizza Hut experienced no significant changes in both level and trend. Papa Johns experienced a significant change in trend (−0.0002, p = 0.029), just as Dominos in trend (0.0002, p < 0.001). McDonalds, on the other hand, experienced a significant change in only level (−0.0060, p = 0.005). Burger King experienced a significant change in both level (−0.0160, p < 0.001) and trend (0.0001, p < 0.001), while Five Guys experienced no significant changes in level and trend. Panera experienced a significant change in only level (−0.0126, p = 0.014). Chipotle experienced a significant change in both level (−0.0170, p < 0.001) and trend (−0.0003, p < 0.001). Jimmy Johns experienced a significant change in only trend (0.0004, p < 0.001), while Five Guys experienced no significant changes in level or trend. Chili’s experienced a significant change in only trend (0.0002, p = 0.032), while Romano’s Macaroni experienced a significant change in level (0.5574, p < 0.001). Olive Garden, Outback Steakhouse and Legal Seafood experienced no significant changes in level or trend in 2016. Uncle Julio’s experienced a significant change in trend (−0.0027, p < 0.001).

## 2018

After eliminating outliers in 2018, Pizza Hut experienced a significant change in only level (−0.01669, p = 0.009), while Papa Johns experienced a change in only trend (0.0005, p < 0.001). Dominos a significant change in level (0.0099, p < 0.001). McDonalds experienced both a change in level (−0.0067, p = 0.008) and trend (0.0001, p < 0.001). Burger King experienced only a significant change in level (−0.0130, p = 0.004). Five Guys experienced a significant change in level (0.0110,p = 0.047) and trend (0.0001, p = 0.014). Chilis experienced a change in both level (−0.0585, p < 0.001) and trend (−0.0004, p = 0.001). Romano’s Macaroni experienced a significant change in level (−0.4218, p < 0.001) and trend (0.0069, p < 0.001), while Olive Garden experienced a significant change in only level (−0.0166, p = 0.005). Uncle Julio’s experienced no significant changes in level and trend in this year (Table A1).

## References

[b0005] Bleich S.N., Soto M.J., Dunn C.G., Moran A.J., Block J.P. (2020). Calorie and nutrient trends in large U.S. Chain Restaurants 2012–2018. PLoS One.

[b0010] Bleich S.N., Moran A.J., Jarlenski M.P., Wolfson J.A. (2018). Higher-calorie menu items eliminated in large chain restaurants. Am. J. Prev. Med..

[b0015] Block J.P. (2018). The calorie-labeling saga—federal preemption and delayed implementation of public health law. N. Engl. J. Med..

[b0020] Block J.P. (2018). The calorie-labeling saga – federal preemption and delayed implementation of public health law. N. Engl. J. Med..

[b0025] Block J.P., Condon S.K., Kleinman K., Mullen J., Linakis S., Rifas-Shiman S., Gillman M.W. (2013). Consumers’ estimation of calorie content at fast food restaurants: cross sectional observational study. BMJ.

[b0030] Cantor J., Torres A., Abrams C., Elbel B. (2015). Five years later: awareness of New York City’s calorie labels declined, with no changes in calories purchased. Health Aff..

[b0035] Cawley J., Susskind A.M., Willage B. (2021). Does information disclosure improve consumer knowledge? Evidence from a randomized experiment of restaurant menu calorie labels. American. J. Health Econ..

[b0040] Cleveland L.P., Simon D., Block J.P. (2020). Federal calorie labeling compliance at US chain restaurants. Obes. Sci. Pract..

[b0045] Elbagir, Shihab, Jing Yang. Twitter sentiment analysis using natural language toolkit and VADER sentiment. *Proceedings of the international multiconference of engineers and computer scientists*. Vol. 122. 2019.

[b0050] Elbel B. (2011). consumer estimation of recommended and actual calories at fast food restaurants. Obesity (Silver Spring).

[b0055] Food and Drug Administration, HHS. (2014). Food labeling; nutrition labeling of standard menu items in restaurants and similar retail food establishments. Final rule. *Federal register*, *79*(230), 71155-71259. 25438344

[b0060] Fryar, Cheryl D., et al. Fast food consumption among adults in the United States, 2013–2016. (2018). 30312154

[b0065] Hswen Y., Moran A.J., Prasad S., Li A., Simon D., Cleveland L., Hawkins J.B., Brownstein J.S., Block J. (2021). The federal menu labeling law and Twitter discussions about calories in the United States: an interrupted time-series analysis. Int. J. Environ. Res. Public Health.

[b0070] Intouch insight (2019, April 1) https://www.intouchinsight.com/blog/10-essential-restaurant-kpis-to-measure-customer-experience-success.

[b0075] Joe M., Lee S., Ham S. (2020). Which brand should be more worried about nutritional information disclosure: McDonald’s or Subway?. Appetite.

[b0080] Kiszko K.M., Martinez O.D., Abrams C., Elbel B. (2014). The influence of calorie labeling on food orders and consumption: a review of the literature. J. Community Health.

[b0090] Mhlanga O., Tichaawa T.M. (2017). Influence of social media on customer experiences in restaurants: a South African study. Tourism..

[b0095] Moran A.J., Roberto C.A. (2018). Health warning labels correct parents’ misperceptions about sugary drink option. Am. J. Prev. Med..

[b0100] Oliver R.L. (1980). A cognitive model of the antecedents and consequences of satisfaction decisions. J. Mark. Res..

[b0105] Petimar J., Ramirez M., Rifas-Shiman S.L., Linakis S., Mullen J., Roberto C.A., Block J.P. (2019). Evaluation of the impact of calorie lableing on McDonald’s restaurant menus: a natural experiment. Int. J. Behav. Nutr. Phys. Act..

[b0110] Petimar J., Zhang F., Rimm E.B., Simon D., Cleveland L.P., Gortmaker S.L., Block J.P. (2021). Changes in the calorie and nutrient content of purchased fast food meals after calorie menu labeling: A natural experiment. PLoS Med..

[b0115] Rao, Tushar, and Saket Srivastava. Analyzing stock market movements using twitter sentiment analysis. (2012): 119-123.

[b0120] Roberto C.A., Larsen P.D., Agnew H., Baik J., Brownell K.D. (2010). Evaluating the impact of menu labeling on food choices and intake. Am. J. Public Health.

[b0125] Rousseeuw P.J., Hubert M. (2011). Robust statistics for outlier detection. Wiley Interdiscip. Rev.: Data Mining Knowledge Disc..

[b0130] Royston P. (1992). Lowess smoothing. Stata Tech. Bull..

[b0135] Syzmanski D.M., Henard D.H. (2001). Customer satisfaction: a meta-analysis of the empirical evidence. J. Acad. Mark. Sci..

